# Supplementary material for: Quorum-Sensing Signal DSF Inhibits the Proliferation of Intestinal Pathogenic Bacteria and Alleviates Inflammatory Response to Suppress DSS-Induced Colitis in Zebrafish
Source: Nutrients. 2024 May 22;16(11):1562. doi: 10.3390/nu16111562 (PMC11173708; doi:10.3390/nu16111562)
Supplement: Supplementary file 1 [file nutrients-16-01562-s001.zip › Suppl.Table S1.pdf]

Table S1: The total number of reads.

Sample	RawPE	Combined	Qualified	Nochime	Base(nt)	Avglen(nt)	GC	Q20	Q30	Effective%
A1	96349	95248	93471	81440	34842780	427.83	53.99%	98.58%	95.04%	84.53%
A2	100451	99706	97897	92830	39735457	428.05	53.93%	98.53%	94.90%	92.41%
A3	87699	86904	85259	77081	32992800	428.03	53.91%	98.62%	95.14%	87.89%
A4	93076	92365	90724	84471	36134419	427.77	53.89%	98.54%	94.88%	90.75%
A5	92644	88410	86333	74448	31682291	425.56	54.15%	98.44%	94.76%	80.36%
A6	87120	86326	84610	78219	33490574	428.16	53.94%	98.54%	94.94%	89.78%
B4	86891	86214	84792	80306	34153170	425.29	54.29%	98.64%	95.17%	92.42%
B5	88087	87294	85712	79622	33794469	424.44	54.35%	98.61%	95.13%	90.39%
B6	92339	91570	89699	81955	34858445	425.34	54.18%	98.42%	94.50%	88.75%
B1	86498	85907	84386	80560	34326877	426.1	54.21%	98.68%	95.32%	93.14%
B2	90430	89700	88109	84197	35889713	426.26	54.08%	98.65%	95.22%	93.11%
B3	92997	92320	90708	85980	36655330	426.32	54.10%	98.70%	95.36%	92.45%
C1	94834	93736	91918	87962	37570834	427.13	54.73%	98.62%	95.13%	92.75%
C2	87849	87173	85731	79450	33914383	426.86	54.75%	98.66%	95.23%	90.44%
C3	88798	87853	86156	82420	35238123	427.54	54.69%	98.65%	95.16%	92.82%
C4	88404	87722	86123	83770	35813484	427.52	54.62%	98.63%	95.16%	94.76%
C5	88490	87802	86216	81336	34749090	427.23	54.70%	98.70%	95.32%	91.92%
C6	87701	86981	85476	80072	34193479	427.03	54.80%	98.65%	95.23%	91.30%
