# Supplementary material for: Quorum-Sensing Signal DSF Inhibits the Proliferation of Intestinal Pathogenic Bacteria and Alleviates Inflammatory Response to Suppress DSS-Induced Colitis in Zebrafish
Source: Nutrients. 2024 May 22;16(11):1562. doi: 10.3390/nu16111562 (PMC11173708; doi:10.3390/nu16111562)
Supplement: Supplementary file 1 [file nutrients-16-01562-s001.zip › Suppl.Table S2.pdf]

Supplementary Table S2: List of all primer sequences.

Zebrafish cDNA primer sequence used for qRT-PCR

Gene	Primer	Sequence
tnfa	forward	AAGGAGAGTTGCCTTTACCG
	reverse	GCCTTGTGAAATGCGATCTCT
il10	forward	GCTCTGCTCACGCTTCTTC
	reverse	CCAAGTCATCGTTGGACTCATA
il6	forward	TGCTACACTGGCTACACTCTT
	reverse	CACATCCTGAACTTCGTCTCC
il1b	forward	ATGGCGAACGTCATCCAAGAG
	reverse	TTCAAGTCGCTGCTTCCGGCT
nfkb1	forward	GGTAGGACTTGCGGTTCTTCT
	reverse	ACCAGCATTCAGATCGCCATC
cox2	forward	TCCAGTACCAGAACCGTATTGC
	reverse	AAGGAGTCCACCATGTTGCG
tab1	forward	AGACAGACAGCAGTGATCTCAG
	reverse	GCTCTGCGATGATTGGCTTATG
fadd	forward	TGAACTCTTCGAGTGCTTGATC
	reverse	AGCTGCTCAACCACTACTTCG
occludin1	forward	CTGCTTCATGGATCCTGAGGAG
	reverse	CCAGTCTTGTGAATTCTGGCTCC
claudin1	forward	GCTGGGTTACTGTCTGGGTTT
	reverse	CCTGTGCTGTGATGATGTTGTC
actin	forward	GGCTACAGCTTCACCACCA
	reverse	TGCTGATCCACATCTGCTG
